# The effect of miniaturized manual versus mechanical instruments on calculus removal and root surface characteristics: An in vitro light microscopic study

**DOI:** 10.1002/cre2.218

**Published:** 2019-07-15

**Authors:** Fabia Profili, Scilla Sparabombe, Andrew Tawse Smith, Orlando D'Isidoro, Alessandro Quaranta

**Affiliations:** ^1^ Private Practice Osimo Italy; ^2^ School of Dental Hygiene Università Politecnica delle Marche Ancona Italy; ^3^ Department of Oral Sciences, Faculty of Dentistry University of Otago Dunedin New Zealand; ^4^ Private Practice Silvi Marina (TE) Italy; ^5^ Discipline of Periodontics and Implantology, School of Dentistry and Oral Health Griffith University Gold Coast QLD Australia

**Keywords:** calculus, nonsurgical periodontal therapy, periodontitis, scaling and root planing

## Abstract

**Objectives:**

The aim of this study was to evaluate by light microscopy analysis the effect of the use of miniaturised piezoelectric tips versus mini‐five area specific curets on calculus removal and postoperative root surface alterations.

**Methods:**

A total of 20 extracted teeth were used. Two square surfaces (5×5 mm) were marked on each root surface with a diamond bur mounted on a high‐speed handpiece. Before and after instrumentation, a series of magnified images (4.2×) of each experimental surface were taken with a standardized approach. According to a randomization list, the two surfaces on each sample were instrumented in a standardised fashion either with a mine‐five curet or a slim piezoelectric tip. The images were processed using an imaging software. Data were summarised as means and standard deviations for the two outcomes (calculus and alterations.) at each time (pre and post) for both of the groups (manual and mechanical).

**Results:**

Both manual and mechanical instrumentation significantly reduced the calculus deposits (*p* < .001) without significant differences between the two groups. Both manual and mechanical treatments significantly increased alterations (*p* < .01). There was a statistically significant evidence of a greater increase in alterations from mechanical treatment.

**Conclusions:**

Slim mechanical piezoelectric tips and manual mini‐five area‐specific curets have similar effects on calculus removal. Manual instrumentation results in a more homogeneous postoperative root surface with less root alterations.

## 1 INTRODUCTION

Periodontitis is an infective disease provoked by an array of periodontal pathogens inducing dysregulation of immune and inflammatory responses in host periodontal tissues, causing periodontal attachment loss (Kornman, Page, & Tonetti, 1997; Page, 1991).

The removal of the supra and subgingival biofilm and its biological products is the main goal of the initial phase of periodontal therapy (Plessas, 2014), and it can be achieved via manual or ultrasonic instrumentation.

With this regard, it may be helpful to remind some definitions in order to fully comprehend the concept behind the nonsurgical therapy:

Debridement: instrumentation for disruption and removal of the microbial biofilms (Kieser, 1994);
Scaling: instrumentation of the crown and root surfaces of the teeth to remove plaque, calculus, and stains from these surfaces (American Academy of Periodontology, 2001a);
Root planing: a treatment procedure designed to remove cementum or surface dentin that is rough, impregnated with calculus, or contaminated with toxins or microorganisms (American Academy of Periodontology, 2001b);

Because periodontitis is strongly associated with the presence of bacterial biofilm and dental calculus on the root surface, the ultimate goal of non surgical therapy is to render the root free from microbial deposits and calculus.

In the past, it was suggested that removal of plaque, calculus, and cementum contaminated with bacterial products and components (e.g., endotoxin) was required by thorough scaling and root planing to achieve periodontal health (Zappa et al., 1991).

Nabers proposed the removal of softened and contaminated root surface in order to obtain a smooth and hard surface able to promote the formation of a new long junctional epithelium (Nabers, 1970). Other authors promoted root planing as able to render diseased root surfaces approximately as free of detectable endotoxin as uninvolved, healthy root surfaces (Jones & O'Leary, 1978).

However, it has been shown that it is not feasible to remove all deposits, and yet despite this it is still possible to achieve a good clinical outcome with nonsurgical therapy. This indicates a threshold level of bacteria, below which the host is able to cope with the remaining infection.

Some additional findings lead to the current perspective that cementum does not need to be removed for a good therapeutic outcome (although practically difficult during calculus removal), because endotoxins weakly adhere to root surfaces without penetration into cementum (Hughes & Smales, 1986; Nakib, Bissada, Simmelink, & Goldstine, 1982; Nyman, Westfelt, Sarhed, & Karring, 1988).

This has been also confirmed by Moore et al. that showed that 99% of lipopolysaccharide (LPS) can be removed by gentle procedures, that is, washing in water and brushing (Moore, Wilson, & Kieser, 1986).

Therefore, the goal of periodontal treatment is to reduce the bacterial load below this level and to modify those aspects of the host response, which can be altered to achieve a more favourable outcome (smoking, diabetes, poor oral hygiene, and stress; Genco & Williams, 2010; Cercek, Kiger, Garrett, & Egelberg, 1983).

Periodontal therapy can be performed with manual and machine‐driven instruments. There is no clinical or experimental evidence about the superior effectiveness in debridement of hand versus mechanical instrumentation (Cobb, 1996; Krishna & De Stefano, 2016; Leknes, Lie, Wikesjo, Bogle, & Selvig, 1994; Sohail Zafar, 2016). Powered scalers utilized in mechanical debridement procedures are classified into sonic and ultrasonic instruments according to their working frequencies. Frequency or speed and amplitude or length of the stroke are the parameters to consider in the choice of ultrasonic scalers. The power setting of the device can be set by the clinician and determines the length (or amplitude). Chapple et al. analysed the effectiveness of the scaling procedure in relationship to the amplitude of the stroke. The results show that the use of the half power setting was as effective as using the ultrasonic scaler at full power (Chappie, Walmsley, Saxby, & Moscrop, 1995). Higher power settings, on the other hand, increase the chipping action, which not only removes calculus but can damage root structure (Lea, Landini, & Walmsley, 2003). Also, the tip displacement amplitude at a medium power setting of 5 or 6 is higher with piezoelectric scalers than with magnetostrictive units.

The piezoelectric scalers use electrical energy to electrosize crystals housed within the handpiece. The dimensional changes of these crystals cause the generation of high vibrational energy that travels to the tip (Flemmig, Petersilka, Mehl, Hickel, & Klaiber, 1998a). Several studies concluded that because of the technology itself, piezoelectric devices create a higher degree of root damage compared with magnetostrictive scalers running at the same power setting (Busslinger, Lampe, Beuchat, & Lehmann, 2001; Flemmig, Petersilka, Mehl, Hickel, & Klaiber, 1998b). The frequency is directly proportional to the energy and inversely proportional to the active area of the tip. The greater the frequency, the higher the energy output but the smaller the active area of the tip. A lower frequency, of 25 kHz, results in an active area of 4.3 mm at the terminal tip, whereas a higher frequency, of 50 kHz, will result in an active area of only 2.3 mm. At low frequency under a load of 25 g, the active area of the tip is increased, allowing deeper pocket depths to be reached with diminished generation of heat, thus preventing thermal damage and patient discomfort (Drisko et al., 2000)**.**

The metal stack in the magnetostrictive scaler generates heat, and to prevent overheating, it requires plenty of irrigation during scaling. It is recommended that the flow rate be at least 20–30 ml/min to prevent a temperature increase of more than 5°C that could potentially damage the pulp and dentin. On the other hand, piezoelectric devices do not generate much heat and require less irrigant; however, the cooler water might cause more sensitivity during the procedure (Nicoll & Peters, 1998).

As it was mentioned above, one of the goals of periodontal therapy is the removal of mineralized deposits and biofilm. The role of calculus and their pathogenicity has been studied. Several studies show that calculus does not induce inflammation in itself. Listgarten and Ellegaard 1973 showed that epithelium can attach to calculus following disinfection with chlorexidine (Listgarten & Ellegaard, 1973). Allen and Kerr (1965) showed full connective tissue encapsulated around autoclaved calculus in guinea pigs (Allen & Kerr, 1965)**.** These studies all show that calculus alone does not induce inflammation. On the other hand, calculus provides a surface which is conductive to plaque accumulation. So the rationale for calculus removal is related to remove it as it is a local predisposing factor for biofilm accumulation.

In addition to it, clinical studies showed that the degree of gingival healing, that was evaluated from cumulative frequency of bleeding, is related to the presence of residual calculus as clinically determined, but not to calculus observed microscopically (Sherman, Hutchens, & Jewson, 1990) _._

Moreover, the same authors estimated that the average surface area with residual microscopic calculus was 5% and that represented a tenfold reduction compared with untreated control surfaces (Sherman, Hutchens, Jewson, Moriarty, et al., 1990)_._

As seen so far, there are no differences in efficacy of deposit removal between manual versus mechanical treatment. The two main differences between these the two instrumentation modalities are probably in dental hard tissue removal and in the increase of the root surface roughness. The manual instrumentation leaves behind a smoother surface, but the ultrasonic has shown to be more conservative in terms of root surface removal (Benfenati, Montesani, Benfenati, & Nathanson, 1987; Suppipat, 1974). For this reason, hand instrumentation has been recommended to smooth the root surface after ultrasonic use as a final finishing procedure in the treatment of periodontitis‐affected roots (Ruppert, Cadosch, Guindy, Case, & Zappa, 2002). With this regard, a sharpened and cutting edge and performing manufacturing materials are recommended as well (Krishna & De Stefano, 2016; Sisera, Hofer, Sener, Attin, & Schmidlin, 2009)**.**

Ritz et al. determined the amounts of root substance removed by four different methods of instrumentation, hand curet, ultrasonic scaler, air scaler, and fine grit diamond bur. Measurement of tooth substance loss was carried out with a specially constructed measuring device at 360 sites on 90 mandibular incisors following 12 working strokes with a clinically appropriate force of application. Only a thin layer of root substance (11.6 μm) was removed by the ultrasonic scaler, compared with the much greater losses sustained with the air‐scaler (93.5 μm), the curette (108.9 μm), and the diamond bur (118.7 μm; Ritz, Hefti, & Rateitschak, 1991)**.**

Although several studies had evaluated the impact of manual versus mechanical instrumentation on root surface characteristics, the results are inconclusive (Jotikasthira, Lie, & Leknes, 1992; Tunkel, Heinecke, & Flemmig, 2002; Wilkinson & Maybury, 1973) and it is not clear whether root surface roughness is more or less pronounced following power‐driven or manual instrumentation.

In addition, most of the studies have been carried out with standard design curets and mechanical tips (Kerry, 1967; Moskow & Bressman, 1964; Stendhe & Schaffer, 1961).

Therefore, the aim of this study was to evaluate by light microscopy analysis the effect of periodontal instrumentation on extracted teeth with slim piezoelectric tips versus miniaturized area‐specific curets in terms of calculus removal and root surface alterations.

## 2 STUDY POPULATION AND METHODOLOGY

Twenty single‐rooted and multirooted teeth were collected after extractions performed at the Umberto I Hospital in Ancona, Italy, Azienda Ospedaliero‐Universitaria Ospedali Riuniti. All the teeth (specimen) were extracted for severe periodontal disease.

Teeth presenting root cavities, root resorption, and fillings over the enamel cementum junction were not used as samples for the present study.

First, the specimen were carefully washed under running water to remove organic material and hematic residues, then they were stored in isotonic solution of sodium chloride at 9% and kept in a refrigerator set at 4–5°C.

After 5 days, the specimen were removed from the sodium chloride solution and dried with high‐pressure air spray.

Two root areas on each tooth were randomly assigned to test (A, manual mini‐five area‐specific curet) and control (B, machine driven slim tip) treatment.

A 1‐mm‐thick rotating stone disk mounted on a high‐speed handpiece was used to create an area of 5 × 5 mm that corresponded to the test or control experimental sites and that was easily identifiable under light microscopy.

A combination of word and numerical codes was used to record and identify each sample and experimental area in a progressive fashion (from 1A to 20A and 1B to 20B).

**Figure 1:**
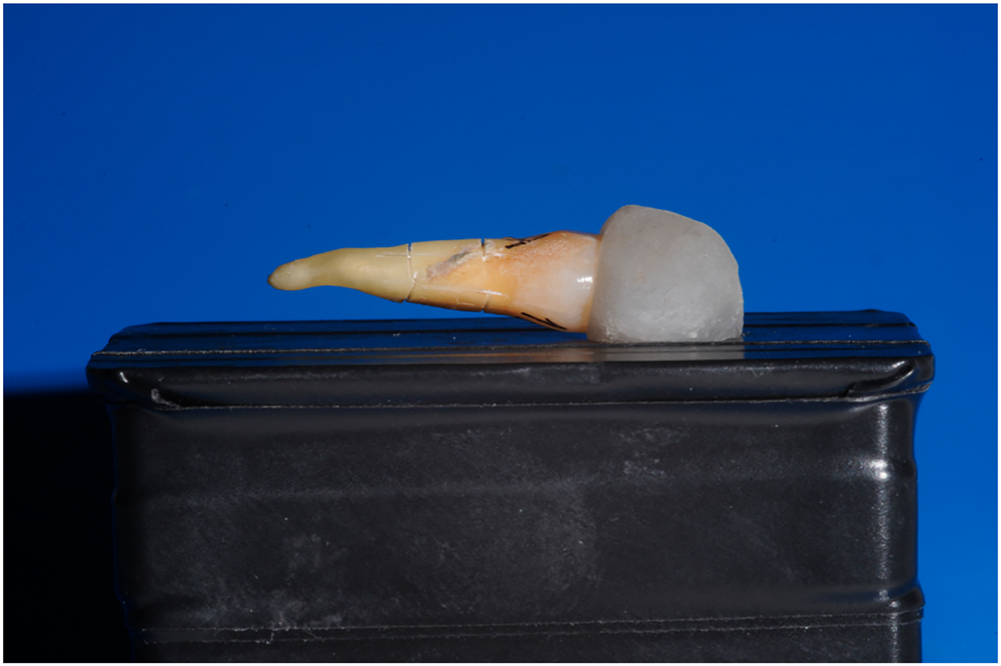
Each sample was mounted on a plastic holder (6‐cm length, 2‐cm height) and secured with orthodontic wax along its longitudinal axis

In order to carry out a standardized photographic analysis, each sample was mounted on a plastic holder (6‐cm length, 2‐cm height) and secured with orthodontic wax along its longitudinal axis (Figure 1).

The plastic support was placed parallel to the lens and perpendicular to the microscopy focal axis.

**Figure 2:**
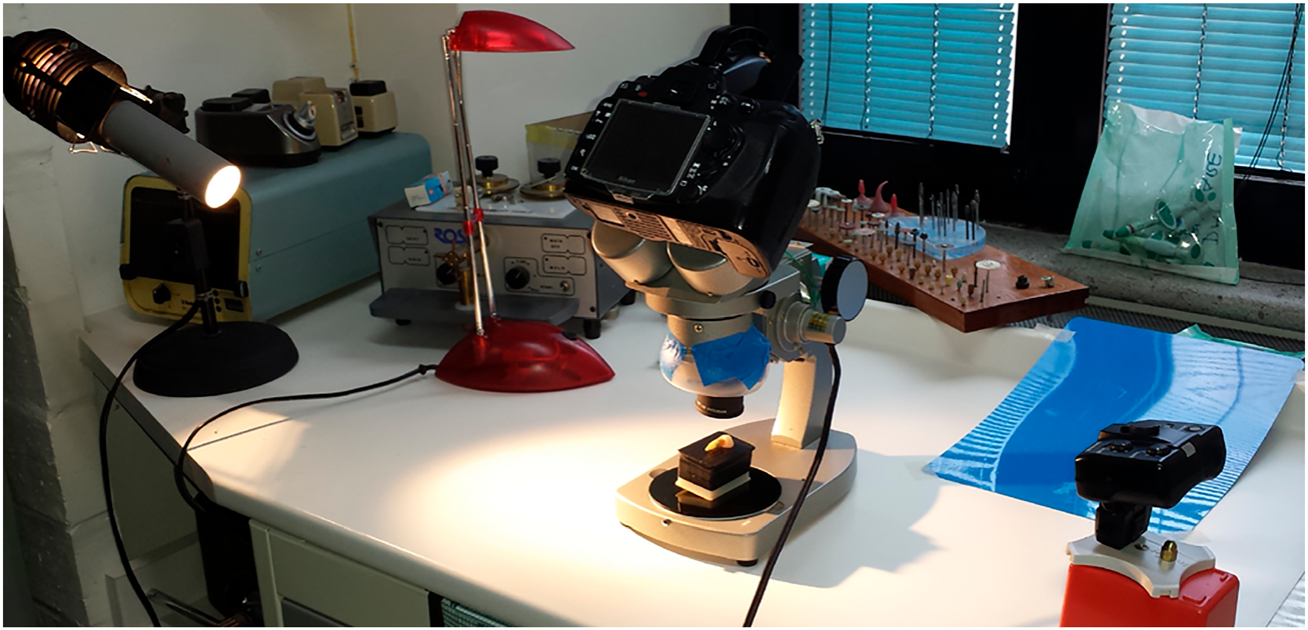
Digital camera (Nikon D300, Nikon Flash SB‐R200, Japan) connected to the light microscopy (ADMIRAL, G.M.C., Switzerland; 2X magnification power)

Standardized pictures were taken for all the 40 specimen before and after the instrumentation procedures using a digital camera 1Nikon D300, Nikon Flash SB‐R200, Nikon Corporation, Japan. that was connected to the light microscopy 2Admiral G.C.M., Switzerland. (2X magnification power; Figure 2).

Automatic control of the light intensity and diffusion was adopted, and the camera was activated using a 80‐cm cable with an electronic shutter click, so to avoid any undesired movement and out of focus pictures.

The images were captured at a 12.3 megapixel resolution. The frame width was 5.6 mm and the sensor width was 23.6 mm. The image final conversion from the frame width to the sensor produced a magnification factor of 4.2:1.

All the pictures were captured as “jpg” format (dimensions: 3,216 × 2,136 pixel).

From descriptive analysis of the 40 tested surfaces before the instrumentation, it was possible to assume that there was homogeneity in deposit amount and irregularities on the surfaces at the baseline with more than 50% of the surfaces free from irregularities in both groups.

All the instrumentation procedures were carried out by a single experienced dental hygienist (S.S.) with the use of Galilean magnifying loupes (2X).

The manual test sites (A) were instrumented, using a mini‐five Gracey curet 3Hu‐Friedy 7/8SAS7/8R9, Hu‐Friedy Mfg.Co., USA. and performing 25 homogeneous apico‐coronal strokes while keeping the terminal shank as much parallel as possible to the tooth long axis.

The control areas (B) were mechanically instrumented with a piezoelectric unit 4Air‐Flow Master, EMS, Switzerland. that mounted a slim debridement tip. 5EMS PS, EMS, Switzerland. The tip was kept as much parallel as possible to the tooth long axis, and a series of light horizontal apico‐coronal movements with light lateral pressure were performed under abundant irrigation. The working time for each mechanical specimen was 30 s, and the power was set on a value of 5 in a scale from 1 to 10.

Once the instrumentation procedures were completed, each specimen was first cleaned with distilled water and then dried with light air‐pressure jet to remove any remaining non attached calculus residual and smear layer, and the photographic analysis was carried out as described above.

A qualitative and quantitative assessment of the images was made independently by three authors not involved in the instrumentation phase (F. P., A. Q., and A. T. S.).

All the pictures were analysed before and after the different procedures using an imaging software 6Preview, Apple Inc., Cupertino, USA. and displaying the images on a wide high‐definition desktop computer screen.

**Figure 3:**
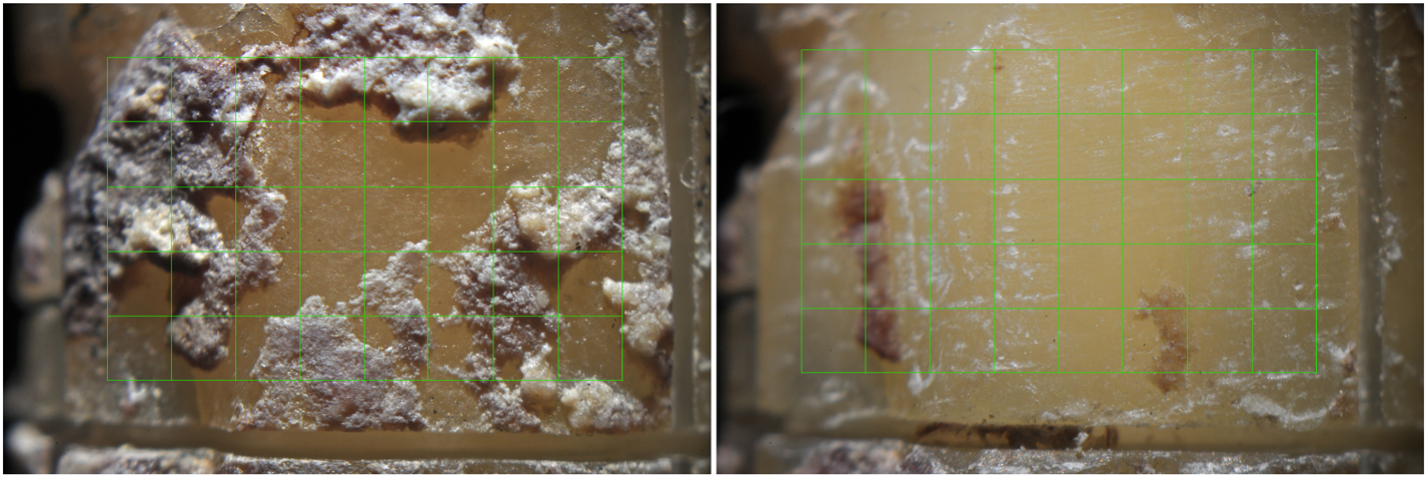
A rectangular grid composed of 40 equivalent squares (height: 5 squares; length: 8 squares) was superimposed to each image

For this purpose, a rectangular grid composed of 40 equivalent squares (height: 5 squares; length: 8 squares) was superimposed to each picture (Figure 3).

This allowed to quantify the number of squares with calculus residuals out of the total number of squares before and after the instrumentation procedures.

Any evident groove, scratch, cavity, demineralized, and/or porous areas that were visible on the screen after the instrumentation was considered as a surface alteration and assessed before and after the instrumentation procedures.

For this purpose, a quantitative analysis of the irregularities was carried using a modification of a previously published scale. This scale ranged from 0 to 3 (Morgan & Marshall, 1998):

0 = no obvious irregularities, resulting in an essentially glass‐like surface;
1 = faint irregularities noticeable, but surface was essentially smooth;
2 = distinct irregularities visible, resulting in a visibly roughened surface; and
3 = gross irregularities of exaggerated depth with a distinct roughened, irregular surface.

The scale was adjusted to the calculated values, providing a variability within 20% of the observed surface.

For the statistical analysis, data were summarized as means and standard deviations for both outcomes (calculus and surface irregularities) at each time (pre and post) for every group (manual and mechanical). For both calculus and surface alterations measures, values from the three raters were averaged together to give a single estimate for each surface at each time. As both outcomes were overdispersed, negative binomial regression was used rather than Poisson regression to model the number of squares (between 0 and 40) scored as positive for calculus or irregularities. For between group comparisons, postmeasurements were compared adjusting for premeasurements. As for within group tests, mixed effects negative binomial regression was used with a random surface id effect to accommodate the paired data. All statistical analyses were performed using a specific software, 7Stata 13.1, StataCorp LLC, USA. and two‐sided *p* < .05 was considered statistically significant in each case. The descriptive analysis was completed by means of synthesis diagrams.

## 3 RESULTS

Changes in deposits amount and irregularities have been observed in both groups after instrumentation (Table 1).

**Table 1:** Premeasurement and postmeasurement of outcomes by group

Group	Calculus						Irregularities					
	Pre	Post	Change within group		Difference between groups		Pre	Post	Change within group		Difference between groups	
	Mean (SD)	Mean (SD)	IRR (95% CI)	p‐value	IRR (95% CI)	p‐value	Mean (SD)	Mean (SD)	IRR (95% CI)	p‐value	IRR (95% CI)	p‐value
Mechanical	24.8 (12.8)	3.7 (3.2)	0.15 (0.11–0.20)	<.001	0.60 (0.35–1.03)	.063	0.5 (0.6)	4.6 (3.1)	9.2 (4.8–17.7)	<.001	Ref	
Manual	30.3 (10.8)	7.1 (8.3)	0.21 (0.14–0.32)	<.001	Ref		0.4 (0.6)	6.1 (3.5)	17.3 (8.1–37.0)	<.001	1.42 (1.04–1.94)	.027

Abbreviations: CI: confidence interval; IRR: incidence rate ratio; SD: standard deviation.

**Figure 4:**
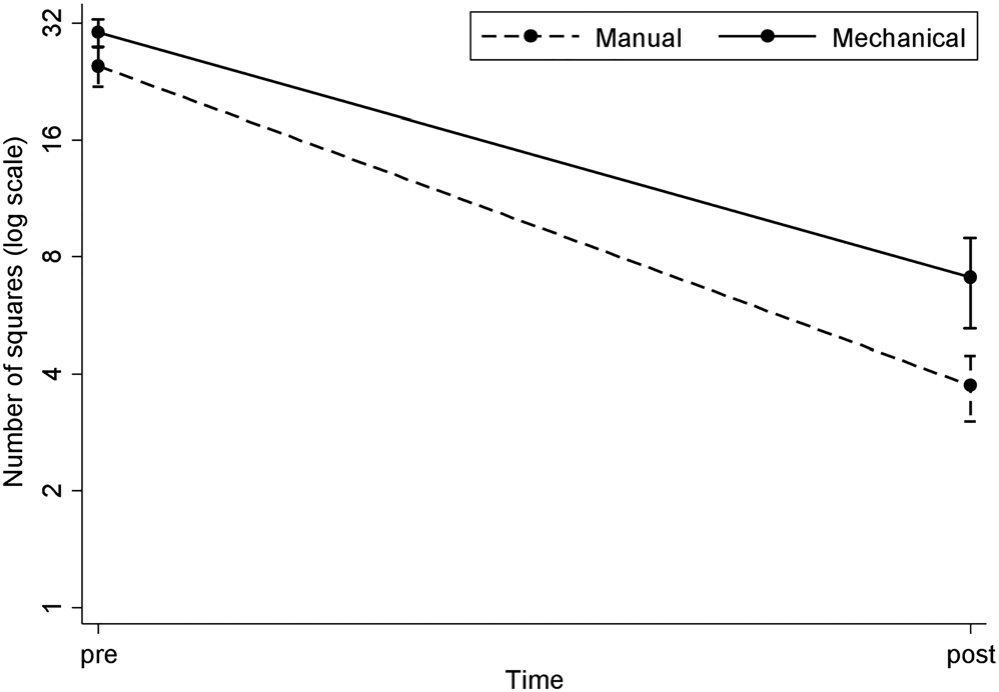
Calculus removal (showing mean ± SD, note log scale on y‐axis)

Both manual and mechanical treatments significantly reduced the calculus deposits on the treated areas (*p* < .001). There was evidence of a greater reduction in calculus from the manual treatment without any statistical difference (*p* = .063; Figure 4).

**Figure 5:**
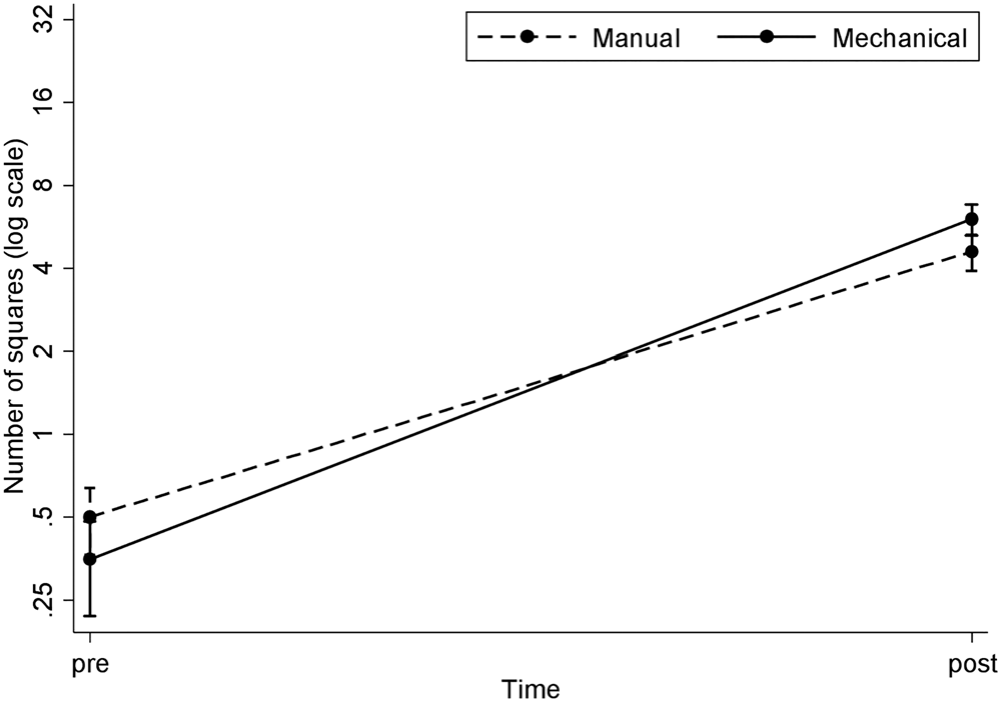
Surface irregularities (showing mean ± SD, note log scale on y‐axis)

Both manual and mechanical treatments increased surface irregularities. (*p* < .01). More specifically, there was statistically significant evidence of a greater increase in irregularities from the mechanical treatment (42% higher at follow‐up, *p* = .027; Figure 5).

The graph (Figure 5) also confirms that there were more surface alterations (expressed as percentages of compromised surfaces) in the group treated with ultrasonic instruments compared with the group treated manually.

## 4 DISCUSSION

The main goal of nonsurgical treatment of periodontitis is the removal of biofilm calculus deposits and bacterial toxins from root surface in order to obtain a biological structure on which a new cellular adhesion can be achieved (Breininger, O'Leary, & Blumenshine, 1987; Rateitschak‐Plüss et al., 1992). The complexity of anatomical structures, dental topography, and the scarce visibility make the periodontal treatment hard to be performed and its clinical results on the root surface difficult to evaluate (Brayer, Mellonig, Dunlap, Marinak, & Carson, 1989).

Although the emphasis of contemporary nonsurgical periodontal therapy is the control of biofilm and plaque retentive calculus to obtain a favourable tissue response, it is extremely challenging in the everyday clinical practice to perform the instrumentation as three separate stages of treatment (debridement, scaling, and root planing). It is therefore essential to select the instruments and the instrumentation pattern that minimizes the risk of having morphological alterations on the root surface and excessive cementum removal (Fleischer, Mellonig, Brayer, Gray, & Barnett, 1989).

In fact, although in the past it was believed that endotoxins were able to adhere deeply to the exposed cementum surface, it has been showed more recently that both bacteria and endotoxins on the cementum can be removed by polishing. In addition to it, despite endotoxins may penetrate in the cementum, they can be easily neutralized by the immune host response (Nyman, Sarhed, Ericsson, Gottlow, & Karring, 1986).

Although the exact amount of endotoxin penetration into cementum and dentin is still unknown, in vitro studies have demonstrated that gingival fibroblasts are not viable on contaminated root surfaces (Aleo, De Renzis, & Farber, 1975; Ramfjord, 1980).

As a result, it is still not clear to which extent intentional removal of root cementum is desirable. Many authors have shown that in order to obtain a smooth and cleaned surface, a minor amount of dental tissue should be removed (Ciantar, 2014; Coldiron, Yukna, Weir, & Caudill, 1990; Mengel, Stelzel, Mengel, Flores‐de‐Jacoby, & Diekwisch, 1997).

Several studies have confirmed the effectiveness of mechanical ultrasonic instrumentation in deposit and endotoxin removal from the root surfaces of periodontally compromised teeth, showing that it is possible to obtain a biologically compatible surface for the new periodontal attachment (Breininger et al., 1987; Copulos, Low, Walker, Trebilcock, & Hefti, 1993; Drisko, 1993).

In addition to biofilm and calculus removal, another important aspect to take into account is the residual roughness of root surface after periodontal instrumentation (Aleo & Vandersall, 1980; Bye, Ghilzan, & Coffesse, 1986).

Although in vivo studies shown that roughness has a minimum impact on periodontal healing and the building of a new periodontal attachment, it can facilitate plaque accumulation and subsequent development of a complex biofilm and mineralized deposits (Khatiblou & Ghodssi, 1983). An unanswered question about periodontal instrumentation is the duration and the number of strokes necessary to obtain a healthy environment.

There is contradictory information in the scientific literature regarding the correlations between the effective removal of subgingival deposits and the instrumentation duration (Braun, Krause, Frentzen, & Jepsen, 2005; Busslinger et al., 2001).

From this point of view the operator experience and clinical skills are fundamental (Brayer et al., 1989).

In our study the photographic magnification of the treated area allowed to evaluate how different contemporary instruments perform. The authors aimed to obtain high‐quality macrophotography images on which assessing qualitative and quantitative analyses of standardized instrumentation modalities. Although all the surfaces have been instrumented in ideal standardized conditions of visibility and time (30 s) for a limited marked area (roughly 25 mm), some deposits could still be detected after both the instrumentation modalities.

This confirms the results of other studies that have shown that a complete removal of the biofilm and calculus is overambitious (29–30; 60).

Our microscopic analysis shows that mini‐five area‐specific curettes are optimal instruments for root instrumentation in terms of both calculus removal and surface alterations.

This is in contrast with other studies that compared standard area‐specific curets versus standard tip mounted on machine‐driven instruments and that showed that curets can create deep grooves and remove sound tooth substance from tooth surface when compared with machine‐driven instruments (Santos, Pochapski, Leal, Gimenes‐Sakima, & Marcantonio, 2008).

In addition, our results confirm that manual instrumentation with curettes results in a more homogenous and smoother root surface.

Within the limits of the present in vitro study, slim mechanical piezoelectric tips and manual mini‐five area‐specific curets have similar effects on calculus removal.

Manual instrumentation with mini‐five curets results in a more homogeneous postoperative root surface.

A combination of manual and mechanical approach may still be considered the best instrumentation methodology.

## CONFLICT OF INTEREST

The authors declare that they do not have any conflict of interest in relation to this project.

## SOURCE OF FUNDING

No financial support has been received for this study.
